# Supplementary material for: The natural sequence of events in larval settlement and metamorphosis of Hydroides elegans (Polychaeta; Serpulidae)
Source: PLoS One. 2021 May 13;16(5):e0249692. doi: 10.1371/journal.pone.0249692 (PMC8118285; doi:10.1371/journal.pone.0249692)
Supplement: S1 Table — Measurements were made on one or two larvae from each of 10 larval cultures for each substrate type. (DOCX) [file pone.0249692.s001.docx]

**S1 Table. Mean time and range, in minutes, to the start of the settlement**

**and metamorphosis events in Hydroides elegans on three substrates. Measurements were made on one or two larvae from each of 10 larval cultures for each substrate type.**

| **Event** | **Wild BF** | ***P. luteoviolacea* BF** | **Tail Arrs** |
| --- | --- | --- | --- |
| Slow swimming | Immediate | Immediate | Variable |
| Stopped swimming, circling | 1 (1-12) | 9 (7-9) | 3 (1-6) |
| Begin secreting primary tube | 11 (3-18) | 13 (9-17) | 13 (**0**-20) |
| Begin shedding prototroch cilia | 13 (11-23) | 17 (12-23) | **11** (6-19) |
| Collar deflects anteriorly | after cilia are shed | after cilia are shed | **never** |
| Begin shedding food-groove cells | 25 (15-35) | 32 (24-39) | 19 (10-33) |
| Loss of prototroch cells | never | never | 13 (0-15) |
| Eversion of “toes” posteriorly. | 33 (24-42) | 39 (37-42) | none |

BF, biofilm; Tail Arrs, tailocin arrays from *P. luteoviolacea*, called MACs by Shikuma et al. [10].
